# Industrial Wood Dyes Removal from Aqueous Solutions by Multifunctional Carbons Derived from Polyacrylonitrile

**DOI:** 10.3390/molecules30163391

**Published:** 2025-08-15

**Authors:** Lidia Domínguez-Ramos, Ismael Tejado, M. Sonia Freire, Diego Gómez-Díaz, Massimo Lazzari, Julia González-Álvarez

**Affiliations:** 1 Center for Research in Biological Chemistry and Molecular Materials (CiQUS), Universidade de Santiago de Compostela, 15782 Santiago de Compostela, Spain; lidia.dominguez.ramos@rai.usc.es (L.D.-R.); massimo.lazzari@usc.es (M.L.); 2 School of Engineering, Department of Chemical Engineering, Universidade de Santiago de Compostela, 15782 Santiago de Compostela, Spaindiego.gomez@usc.es (D.G.-D.); julia.gonzalez@usc.es (J.G.-Á.); 3 Faculty of Chemistry, Department of Physical Chemistry, Universidade de Santiago de Compostela, 15782 Santiago de Compostela, Spain

**Keywords:** porous carbons, polyacrylonitrile, tunable pore, red dye, adsorption

## Abstract

Wastewater containing synthetic dyes harmful to aquatic environments supposes significant challenges for treatment. This study focuses on how structural characteristics of three N-containing carbons synthesized at high temperatures from polyacrylonitrile (PAN) as a precursor, i.e., an N-doped (PAN-C), an activated carbon (PAN-C-Act), and a carbon also incorporating sulfur (PAN-S-C), influence adsorption of a common dye employed for wood veneers (Red GRA 200%). The impact of pH (1.9–2.3, 6.0–6.8, and 11.8–12.6), adsorbent dosage (S/L, 0.43–0.53 and 1.73–1.91 g L^−1^), and amount of dye (24–28 mg L^−1^ and 231–285 mg L^−1^) on dye removal from aqueous solutions were investigated. In general, the results obtained in the present work indicate that the presence of larger pores in the materials plays an important role in dye adsorption by preventing size exclusion of the dye molecules. The activated carbon (PAN-C-Act) demonstrated the greatest adsorption performance, with an adsorption yield close to 100% achieved at a carbon dose of 0.47 g L^−1^ and acidic pH for the highest dye concentration and longest experiment time. The pseudo-second-order model best described the kinetics, and both external mass transfer and intra-particle diffusion were confirmed.

## 1. Introduction

Water pollution is an actual issue that is increasing daily [1,2]. Therefore, the presence of harmful compounds such as metals, dyes, and pharmaceuticals in untreated industrial effluents that end up in our rivers, lakes, and oceans represents a serious environmental problem. Numerous process industries across various sectors generate substantial amounts of colored wastewater [3]. In particular, dyeing and finishing processes in the textile industry are major contributors to wastewater pollution, discharging significant amounts of colored compounds [4], and often presenting high levels of suspended solids, organic matter, pH changes, and other inorganic contaminants. The existence of color reduces aquatic biodiversity by interfering with light transit through the water, consequently avoiding the photosynthesis of aqueous flora [5]. In some cases, dye concentrations below 1 mg L^−1^ are enough to cause a noticeable water coloration in water. Also, most of these compounds can cause dermatitis [6] and are toxic, carcinogenic, and mutagenic to human beings [7,8]. Consequently, different treatments of effluent-containing dyes have been developed to mitigate their adverse environmental effects and health risks [5].

In this way, various physical and physicochemical processes have been widely used for dye removal [5,9,10]. However, dyes are molecules with complex chemical structures highly resistant to degradation [11]. Among existing technologies, those based on adsorption are the most common and have shown good efficiencies for dye removal [12]. Over the past few decades, many studies have focused on finding the most effective adsorbent materials. In this context, carbon-based adsorbents have been the most extensively used with good results [13]. These materials tend to be hydrophobic or organophilic depending on their surface polarity, which arises from the surface chemical groups. Thus, they are widely used for the adsorption of organic compounds as dyes in water recovery and purification systems. In addition to the surface chemistry of the adsorbent, its internal porous structure is another key factor as it directly affects the material’s capacity to capture dye molecules. Moreover, the precursor used in carbon production plays a critical role because it affects its internal structure. For example, Table 1 shows representative precursors used for carbon production, along with their adsorption capacities for common dyes and pore volumes, observing the frequent use of lignocellulosic materials due to their low cost. However, a major drawback of lignocellulosic precursors is the limited ability to control the internal structure of the resulting carbonaceous matrix. On the other hand, the use of polymers as precursors allows for better control over pore size and provides materials with larger surface areas.

Polyacrylonitrile (PAN) is an excellent precursor for carbon materials with an extraordinary graphitization degree and a high surface area but relatively low porosity. These characteristics are due to forming a tightly stacked structure with a small number of defects during PAN pre-oxidation. Moreover, PAN is a nitrogen-containing polymer, which can contribute to proportionate functionalities in the carbon structure [14,15]. Also, heteroatom doping causes structural changes modifying the carbon physicochemical properties, which are used to provide carbon materials with adjustable functions for different applications [16]. Thus, when sulfur is incorporated into the carbon structures, the resulting nanocarbons exhibit a great specific surface area and controlled pore size [17]. Also, it has been found that sulfur doping provides the carbons of appropriate characteristics to be used as heavy metals or dye adsorbents and supercapacitors [18,19]. In addition, porosity and carbon-specific surfaces obtained through physical or chemical activation and carbon surface chemistry play a significant role in the adsorption process [20,21].

**Table 1:** Dyes adsorption capacities for carbon materials made from different precursors.

Precursor	Activation	Surface Area (m^2^ g^−^^1^)	Dye	Adsorption Capacity (mg g^−^^1^)	Pore Volume (cm^3^ g^−1^)	Source
Banana peel	---	---	Reactive Black 5	26.9	---	[20]
			Congo red	46.7		
Macroalgae (*Ulothrix zonata*)	---	133.2	Malachite green	5306.2	---	[22]
			Crystal violet	1222.5		
			Congo red	345.2		
Rice straws	KOH	1973.0	Methylene blue	527.6	1.131	[23]
			Congo red	44.2		
	H_3_PO_4_	392.6	Methylene blue	34.7	0.463	
			Congo red	67.1		
	CO_2_	214.7	Methylene blue	44.2	0.164	
			Congo red	253.9		
Bamboo	KOH	1896.0	Methylene blue	454.2	1.109	[24]
Sargassum fusiforme	CO_2_	1329	Congo red	234.0	1.2	[25]
H_2_SO_4_-modified celery residue	---	24.93	Congo red	238.09	0.041	[26]
ZnO-modified SiO_2_ nanospheres	---	34.5	Congo red	83.0	0.16	[27]
Zeolitic imidazolate framework-67	---	1388	Congo red	714.3	---	[28]

In this work, three doped, porous carbons manufactured from polyacrylonitrile, N-doped carbon, N-doped activated carbon, and N,S-co-doped carbon (PAN-C, PAN-C-Act, and PAN-S-C, respectively), were used as adsorbents of an industrial wood dye. The aim was to demonstrate how the structural characteristics of these materials influence dye adsorption, and how appropriate selection can help overcome limitations associated with dye size exclusion. In addition, the effect of pH, initial dye concentration and carbon dosage on the adsorption capacity of each carbon was analyzed. Kinetics and diffusion mechanisms were also evaluated.

## 2. Results and Discussion

Adsorbent characterization was performed in a previous work [29]. BET surface areas, S_BET_, of PAN-C, PAN-S-C, PAN-C-Act, and CAC were 36.3, 150.5, 3154.9, and 1059.9 m^2^ g^−1^, respectively, as determined from N_2_ adsorption isotherms at 77 K. The nitrogen sorption isotherms for PAN-C and PAN-S-C carbons were type I isotherms. PAN-C-Act showed a combination of type I and IV isotherms and CAC presented type II isotherm adsorption. XPS analysis determined the presence of nitrogen and sulfur-containing functional groups, showing that the porous carbons fabricated from polyacrylonitrile had N groups in their structure, such as pyrrolic N and pyridinic N, as well as C-N bonds. However, PAN-C-Act lost pyridinic N groups during activation. PAN-C-S also showed S-rich groups, C-SO_2_-C, sulfur, and disulfide bonds. Moreover, the oxidation and activation stages provided a higher formation of carbonyl and hydroxyl groups. CAC showed only C=O and COOH groups.

The presence of OH-groups determines the acid–base character and reactivity of carbon materials. Moreover, the presence of various other surface functional groups makes surface chemistry more versatile compared to other adsorbents [30]. As a result, pollutants adsorption from aqueous solutions is complex and involves many parameters, such as solution pH, ionic strength, as well as solute–solute and solute–solvent interactions [31]. In this way, several series of adsorption experiments using manufactured carbons and commercial ones have been carried out (Table 2).

### 2.1. Effect of Initial PH

**Figure 1:**
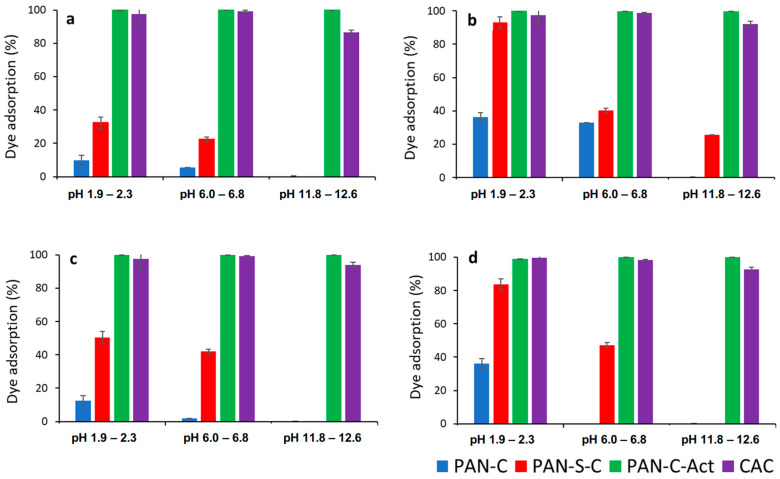
Effect of pH on dye adsorption (%) for PAN-C, PAN-S-C, PAN-C-Act, and CAC. (**a**) C_i_: 24–28 mg L^−1^, S/L: 0.43–0.53 g L^−1^; (**b**) C_i_: 24–28 mg L^−1^, S/L: 1.73–1.91 g L^−1^, (**c**) C_i_: 230–285 mg L^−1^, S/L: 0.43–0.53 g L^−1^; (**d**) C_i_: 230–285 mg L^−1^, S/L: 1.73–1.91 g L^−1^.

Dye solution pH significantly affects the entire adsorption process, particularly the materials’ adsorption capacity [22]. Hence, adsorption of red dye on carbons was studied as a function of pH at acid (1.9–2.3), natural (6–6.8) and alkaline pH (11.8–12.6) in the range of two initial concentrations (24–28 mg L^−1^ and 231–285 mg L^−1^) and carbon dosages essayed (0.43–0.53 and 1.73–1.91 g L^−1^) at 298 K and 48 h. Figure 1 compares the effect of pH on dye removal efficiency for PAN-C, PAN-S-C, PAN-C-Act, and CAC.

As can be observed, pH affected the maximum adsorption efficiency for all carbons, especially for PAN-C and PAN-S-C, and the higher adsorption percentages were obtained at acid pH. Moreover, the PAN-C-Act carbon showed the best removal performance, even better than the commercial one, with an adsorption yield (%) of approximately 100%. Considering the point of zero charge of carbons, at acid and natural pH, lower than pH_PZC_ (8.3 and 8.2 for PAN-C and PAN-S-C, respectively, and 7.4 for both PAN-C-Act and CAC) [29], cationic functional groups predominate on the carbon surface while the surface is negatively charged at alkaline pH. As previously found [32], the dye behaves as a weak acid (pK_a_ of 10.6). Consequently, in aqueous solution, it predominantly exists in its anionic form and is more effectively adsorbed onto cationic surfaces. This behavior has also been evidenced by Al-Degs et al. [33] with a commercial activated carbon F-400. Furthermore, the presence of oxygen functional groups, such as ketone and hydroxyl groups, gives basicity to the carbon surface, with PAN-C-Act showing an increase in the oxygen content after KOH activation with respect to the other two carbons [29]. Conversely, PAN-C is the carbon with the lowest removal efficiency, with a higher yield of 36.2% (5.1 mg g^−1^) at pH 2, adsorbent dosage (S/L = 1.7 g L^−1^), and initial concentration of 24 mg L^−1^. In addition, this carbon has the lower BET surface area (36.3 m^2^ g^−1^) and pore volume (0.018 cm^3^ g^−1^) [29], which can indicate that these characteristics directly influence adsorption capacity. The pH variations observed (Table 2) are due to the solution becoming slightly alkaline as the initial concentration was increased.

### 2.2. Effect of Carbon-Structural Properties

**Figure 2:**
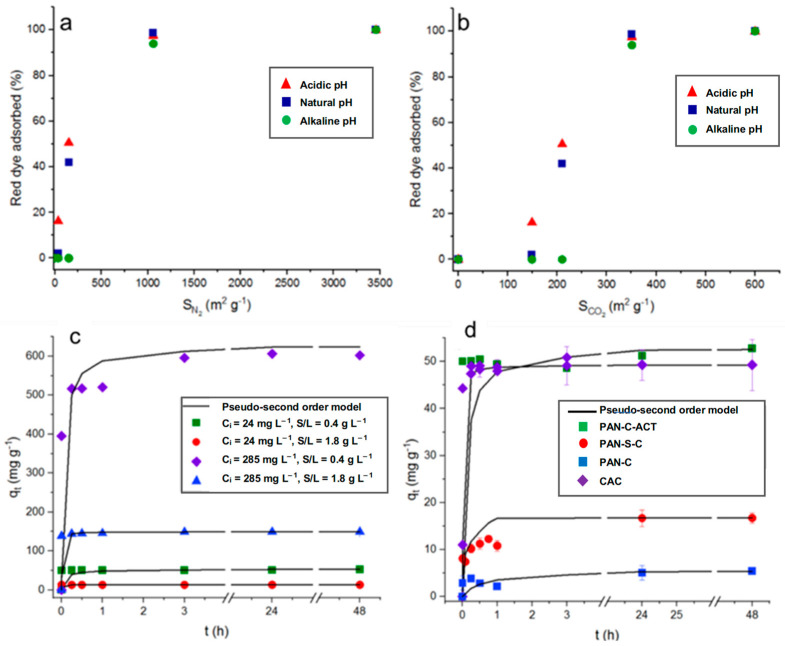
Top plots: Influence of surface area determined in PAN-C, PAN-S-C, PAN-C-Act, and CAC, with (**a**) N_2_ at 77 K and (**b**) CO_2_ at 273 K, on dye adsorption at 48 h (pH: 1.9–2.3; 6–6.8; 11.8–12.6; C_i_: 230–285 mg L^−1^ and S/L, 0.43–0.53 g L^−^^1^). Plots below: Red dye adsorption kinetic data. The lines correspond to a pseudo-second-order model. (**c**) Effect of initial dye concentration (C_i_: 24 and 285 mg L^−^^1^) and adsorbent dosage (0.45–0.47 and 1.86–1.89 g L^−^^1^) on adsorption capacity for PAN-C-Act at pH ~ 2 and 298 K. (**d**) Effect of adsorbent on adsorption kinetics at pH ~ 2 and 298 K (C_i_: 24–28 mg L^−^^1^ and 0.43–0.53 g L^−^^1^).

In general, the materials with a greater surface area tend to adsorb better the molecules under study. To analyze more carefully the effect of the surface area measured by CO_2_ at 273 K and N_2_ at 77 K [29] on dye adsorption, the surface areas of each carbon (PAN-C, 148.7 and 36.3; PAN-S-C, 209.9 and 150.5; PAN-C-Act, 599.7 and 3154.9; and CAC, 350.9 and 1059.9 m^2^ g^−1^, respectively) were related to the red dye adsorption percentages at acid, natural, and basic pH (1.9–2.3; 6–6.8; 11.8–12.6), higher initial concentration (C_i_, 231–285 mg L^−^^1^), and lower carbon dosage (S/L, 0.43–0.53 g L^−^^1^), as shown in Figure 2a,b.

It can be observed that, in general, the adsorption percentage increases with the surface area increasing, regardless of pH. As a result, it was observed in Figure 2a that as the N_2_ surface area tends to very low values, while the adsorption efficiency tends to zero. However, the results in Figure 2b indicate that the CO_2_ surface area does not contribute to the dye adsorption. So, adsorption percentages that are almost nil will be obtained, especially at alkaline pH, even with surface areas of nearly 150 m^2^ g^−1^. From previous studies [34], it was concluded that those pores in the ultramicroporosity range generate the surface area determined with CO_2_ at 273 K (d_p_ < 0.7 nm). Therefore, the results suggest that a part of the surface area is inaccessible for dye adsorption due to a size exclusion phenomenon as the highest distance between two extreme atoms of dye molecule, 1.28 nm [35], exceeds the size of ultramicropores. This fact is very clearly observed for PAN-C (36.3 m^2^ g^−1^ with N_2_ at 77 K and 148.7 m^2^ g^−1^ with CO_2_ at 273 K), in which surface area due to ultramicropores represents 80% of the total area [29], and it is not available for dye access, showing low adsorption percentages under all conditions (Table 2). On the contrary, PAN-S-C, which is also a non-activated carbon but doped with sulfur, showed higher adsorption percentages, with the highest value, 92.0% (*q_max_*, 11.9 mg g^−1^), at pH ~ 2, S/L 1.9 g L^−1^, and 24 mg L^−1^ of initial dye concentration. The presence of S causes the formation of mesopores (68% of total pore volume) [29], allowing the access of dye. As mentioned before, PAN-C-Act with a very large surface area containing high mesopore volume almost completely adsorbed the dye under all conditions essayed (Table 2). From Figure 2a, it is possible to conclude that the results of dye adsorption (%) correlate with the surface area determined by N_2_ adsorption, considering the accessibility of mesopores and larger micropores, while excluding the contribution from ultramicroporosity. This highlights this parameter as a key factor influencing the adsorption performance.

### 2.3. Effect of Initial Concentration and Adsorbent Dosage

The influence of initial dye concentration, which varied from 24 to 28 mg L^−1^ to 231–285 mg L^−1^ depending on the carbon, affected their adsorption performance (Table 2). As it can be observed, for a fixed pH and adsorbent dosage (S/L), as well as a general increase in the dye’s initial concentration, the adsorption capacity of all carbons were up to 70.5, 252.7, 602.3, and 556.1 mg g^−1^ for PAN-C, PAN-S-C, PAN-C-Act, and CAC, respectively, at pH ~ 2, S/L ~ 0.5 g L^−1^, and C_i_ = 231–285 mg L^−1^. This behavior is also shown as a function of time for PAN-C-Act at the initial dye concentrations, and adsorbent doses essayed (Figure 2c).

The adsorbent dosage positively affected the dye adsorption percentage, increasing these values, as shown in Figure 1. If Figure 1a is compared with Figure 1b,c is compared with Figure 1d, a general improvement in the adsorption percentages is observed for PAN-C and PAN-C-S at acid and natural pH, and even at alkaline pH for lower initial concentrations (C_i_, 24–28 mg L^−1^). Increasing adsorbent dosage provides more active sites for the dye to be adsorbed and increases the adsorption percentage, as observed in Table 2. On the contrary, the adsorption capacity of porous carbons decreases with increasing adsorbent dosage, as observed in Table 2 and, specifically, for PAN-C-Act adsorption in Figure 2c. Figure 2d shows the red dye adsorption kinetic data for all carbons and the effect of the type of adsorbent on dye adsorption at 298 K over 48 h (C_i_, 24–28 mg L^−1^ and 0.43–0.53 g L^−1^). The use of PAN-C-Act together with the CAC led to better adsorption performance.

### 2.4. Adsorption Kinetic Modeling

Kinetic studies are important as they provide information on the mechanism of the adsorption process [36]. Figure 2d shows the effect of time on the red dye adsorption capacity of the porous carbons at pH ~ 2, observing fast adsorption of the dye during the first minutes and reaching the maximum adsorption capacity after 24 h, 3 h, and 15 min for PAN-C, PAN-S-C, and PAN-C-Act, respectively, for all experiments performed. Moreover, although the PAN-C-Act reaches a slightly higher dye adsorption capacity than CAC, possibly due to its higher N_2_ surface area joined to its surface chemistry and pore structure, Figure 2d shows that the CAC has a more favorable kinetic. This fact is probably due to the presence of smaller porous PAN-C-Act inside, which exhibits slightly higher microporosity (25.6 vs. 24.4%) and significantly greater ultramicroporosity (599.7 vs. 350.9 m^2^ g^−1^ for CO_2_ surface areas) compared to CAC [29], which significantly decreases the diffusivity of the dye in its porous structure.

Table 2 and Table 3 present the kinetics parameters obtained for the pseudo-second-order and the intra-particle diffusion models, respectively, along with the corresponding determination coefficients. Low R^2^ values indicated that the pseudo-first-order model is inappropriate for fitting the experimental data. On the contrary, the pseudo-second-order kinetic model explained better the adsorption behavior, except for PAN-C at natural pH, obtaining determination coefficients higher than 0.96 (Table 2). The dye diffusion mechanism into PAN-C and PAN-S-C occurs in two simultaneous stages: external mass transfer followed by intra-particle diffusion, as demonstrated by the fitting to the intra-particle diffusion model (Table 3). PAN-C-Act and commercial activated carbon show a two-step adsorption mechanism, where in the first step, there is a fast adsorption of the dye by the carbon. These materials do not fit well to the intra-particle diffusion model since the determination coefficients are often lower than 0.5. In general, a good agreement is observed between the calculated and experimental adsorption capacities for the pseudo-second-order kinetic model (Table 2 and Figure 2c,d).

Adsorption is influenced by various factors, including the adsorbent textural and surface properties, surface functional groups, adsorbate–adsorbent interactions, and the adsorbate diffusion process into the adsorbent. In this work, the results suggest that chemical processes between the dye and the carbon primarily control the overall rate of red wood dye adsorption.

Table 4 shows dye adsorption capacities for commercial activated carbons published by other authors. The activated carbon PAN-C-Act prepared in this work showed, in general, higher adsorption capacity than those commercial ones and those prepared from other precursors (Table 1), though these values are, of course, dependent on the type of dye used and the conditions applied. This comparison underscores the good adsorption capacity of PAN-C-Act and clearly demonstrates the feasibility of synthesizing activated carbons with enhanced properties.

## 3. Materials and Methods

### 3.1. Carbon Synthesis

N-doped carbon (PAN-C), N-doped carbon activated with KOH (PAN-C-Act), and N, S-co-doped carbon with sulfur in a 1:1 weight ratio (PAN-S-C) were prepared using PAN as a precursor (150,000 g mol^−1^), with the experimental procedure reported in the previous work [29] using PAN as a precursor (150,000 g mol^−1^). Briefly, the carbonization process consisted of two continuous stages. The first stage was performed at 553 K to stabilize the PAN structure, initially under an oxygen flow (10 mL min^−1^) for 1 h, followed by 0.5 h under an inert atmosphere at the same temperature. The second stage involved pyrolysis at 1073 K under a N_2_ atmosphere, where the polymer was completely pyrolyzed. For activation, PAN-C was ground with KOH (1:4 *w*/*w*) and carbonized in an inert N_2_ atmosphere at 1073 K (10 mL min^−1^) for 2 h. In addition, commercial activated carbon (CAC) was used as a reference material (Merck, Kenilworth, NJ, USA). Porous carbon characterization, including surface textural properties and morphological features, can be found in the previous work [29].

### 3.2. Wood Red Dye

**Figure 3:**
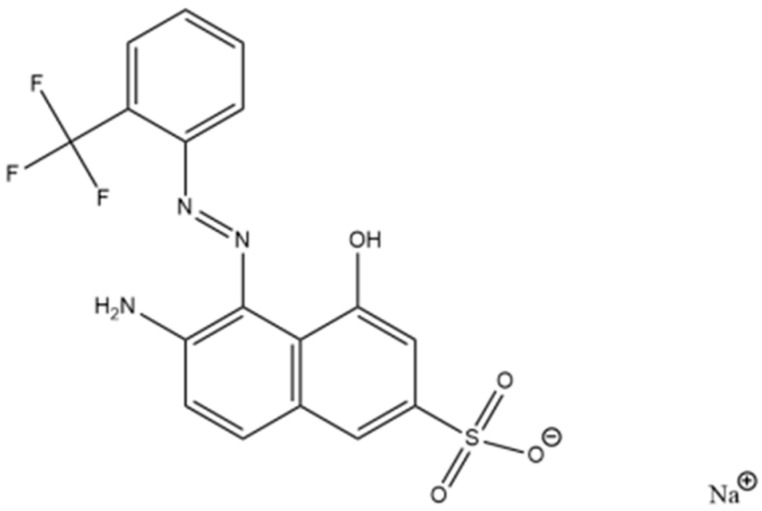
Structural formula of dye red GRA-200% [35].

Red GRA 200% (C_17_H_11_F_3_N_3_NaO_4_S^+^, 433.34 g mol^−1^) is an industrial acidic and anionic wood dye provided by ASERPAL S.A. company (Grupo Losán S.A., Galicia, Spain), which manufactured wood veneer boards. Figure 3 shows the structural formula of the dye. Working solutions at the required red dye concentrations were prepared by diluting an aqueous dye solution of 500 mg L^−1^. Approximately 1 mol L^−1^ NaOH or HCl aqueous solutions (Sigma Aldrich, Steinheim, Germany) were used to adjust pH. A scan of red dye solutions (10 mg L^−1^) between pH 1.5 and 12.5 was performed by UV/VIS spectroscopy (V-630, Jasco, Tokyo, Japan), obtaining the maximum wavelength (λ_max_) at 506 nm for pH from 1.5 to 8. At pH 8, the red dye solution changed to yellow at λ_max_ of 505 nm, and finally, the λ_max_ was 482 nm at pH 11.5. A previous study confirmed that dye solutions under all conditions of initial concentration and pH used were stable over time (48 h). The determination of the acid dissociation constant (pK_a_) of the Red GRA 200% dye was previously performed by a UV–Visible spectroscopic method [32]. pK_a_, which predicts the ionization state of the molecule concerning pH, is 10.6.

### 3.3. Batch Adsorption Experiments

**Figure 4:**
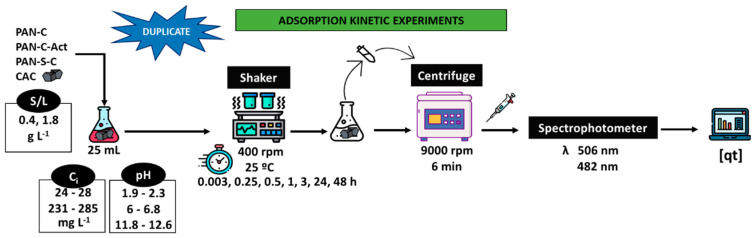
Scheme of the batch adsorption kinetic experiments.

Figure 4 shows the process followed to carry out the adsorption kinetics experiments. Experiments were performed in an orbital mini shaker (VWR, Cienytech, PA, USA) at 25 °C and 400 rpm to study the dye adsorption. The influence of pH (acidic, 1.9–2.3; natural, 6–6.8; alkaline, 11.8–12.6), adsorbent dosage (S/L, 0.43–0.53 and 1.73–1.91 g L^−1^) and initial dye concentration (*C_i_*, 24–28 mg L^−1^ and 231–285 mg L^−1^) were evaluated for over 48 h. At fixed times, the suspensions were centrifuged (Alresa Microcen, Madrid, Spain) at 9000 rpm for 6 min, and the supernatants were collected. Finally, the dye concentration was obtained at the λ_max_. All the experiments were duplicated. A commercial carbon was also used as a comparison.

The adsorption capacity (*q_t_*) in mg g^−1^ was calculated following Equation (1):

(1) $$q_{t}\;=\;\frac{\left(C_{i}-C_{t}\right)\cdot V}{m}$$

where *C_i_* and *C_t_* are the initial dye concentration and concentration at any time *t* (h), respectively (mg L^−1^), *V* is the volume of dye solution (L), and *m* is the dry mass of carbon used (g). The maximum *q_t_* is referred to as *q_max_* and corresponds to the maximum adsorption capacity determined experimentally.

The removal efficiency, expressed as the percentage of dye adsorbed was determined using Equation (2):

(2) $$Dyeadsorbed\left(\%\right)\;=\;\frac{\left(C_{i}-C_{t}\right)}{C_{i}}\cdot 100$$

Lagergren’s first-order model expressed by Equation (3) [43], and Ho’s pseudo-second-order model given by Equation (4) [44] were used to describe dye kinetic adsorption:

(3) $$log\left(q_{e}-q_{t}\right)\;=\;log\left(q_{e}\right)-\frac{k_{1}}{2.303}\cdot t$$

(4) $$\frac{1}{q_{t}}=\frac{1}{k_{2}\cdot q_{e}^{2}}+\frac{1}{q_{e}}\cdot t$$

where *q_e_* is the amount of dye adsorbed on the carbon (mg g^−1^) at equilibrium; *k_1_* (h^−1^) and *k_2_* (g mg^−1^ h^−1^) are the corresponding kinetic constants for the models. The intra-particle diffusion model, Equation (5) [45], was also considered.

(5) $$q_{t}=K_{id}\cdot t^{0.5}+I$$

where *K_id_* and *I* are the intra-particle diffusion rate constant (mg g^−1^ h^−0.5^) and intercept (mg g^−1^), respectively.

## 4. Conclusions

Three porous carbons produced using PAN as a precursor were used as adsorbents of a red wood dye in aqueous solutions. In general, it was found that the adsorption capacity for all carbons increased at acid pH. In addition, the initial dye concentration and carbon dosage considerably affected only the adsorption performance of N-doped carbon (PAN-C) and N,S co-doped carbon with sulfur (PAN-S-C). In the case of N-doped activated carbon (PAN-C-Act), the maximum sorption yield (approximately 100%) was reached for all conditions essayed, with values higher than those obtained for the commercial activated carbon (CAC). In particular, PAN-C-Act showed a higher adsorption capacity (602.3 mg g^−1^) compared to CAC (556.1 mg g^−1^), highlighting its superior performance and greater practical applicability. This achievement was probably due to porosity development and a higher specific surface area. The pseudo-second-order model better fitted the adsorption kinetics, and the existence of external mass transfer followed by intra-particle diffusion was confirmed by the intra-particular diffusion model. In general, the results obtained in the present work conclude that the presence of mesoporosity (>2 nm) in the materials plays an important role in dye adsorption by providing accessible pathways to the dye molecules. In contrast, ultramicroporosity has a negative effect due to molecular size exclusion. Despite the availability of surface area (mainly into the solid particles), adsorption is not attained because dye molecules do not have access to the carbon porous structure. Therefore, the selection of carbons for the red dye removal must be performed based not only on the chemical characteristics of the carbon surface and the total surface area available but also on pore size distribution to avoid dye size exclusion.

## Figures and Tables

**Table 2:** Experimental conditions essayed and kinetic parameters of the pseudo-second-order model for red wood dye adsorption with carbons prepared. * fits better for the first-order model (^a^*q_max_* corresponds to the maximum adsorption capacity determined experimentally, and ^b^*q_e_* is the amount of dye adsorbed (mg g^−1^) at equilibrium according to model fit).

Sample	Experimental Conditions			Adsorbed Dye Percentage (%)	^a^*q_max_* (mg g^−1^)	Pseudo-second-order Model		
	pH	S/L (g L^−1^)	C_i_ (mg L^−1^)			k_2_ (g mg^−1^ h^−1^)	^b^*q_e_* (mg g^−1^)	R^2^
CAC	2.0	0.53	24	97.4	43.7	1.733	43.9	1
	2.0	0.44	250	97.5	556.1	0.032	555.6	0.99
	2.0	1.76	24	97.4	13.3	11.772	13.0	0.99
	2.0	1.91	250	99.8	130.3	0.593	129.87	1
	6.1	0.5	25	99.2	55.2	0.550	54.9	0.99
	6.5	0.46	234	99.1	506.6	0.01	500	0.99
	6.0	1.75	24	98.6	13.5	1.663	13.5	0.99
	6.5	1.74	236	98.2	133.2	1.186	129.9	1
	12.6	0.43	25	86.4	49.9	0.260	49.5	0.99
	12.1	0.45	259	93.9	545.8	0.012	526.3	0.99
	12.6	1.74	26	92.1	13.8	15.093	12.9	0.99
	12.1	1.78	260	92.6	135.0	0.047	133.3	0.99
PAN-C	2.0	0.44	24	9.9	5.4	0.351	5.4	0.99
	2.3	0.45	256	12.5	70.5	0.008	67.1	0.96
	2.0	1.70	24	36.2	5.1	0.254	4.9	0.98
	2.0	1.87	256	35.8	49.0	0.029	47.8	0.99
	6.0	0.44	24	5.5	0.3	0.307	2.3	0.99
	6.5	0.48	231	2.0	9.6	—	—	—
	6.1 *	1.87	24	32.8	4.2	0.06 * k_1_ 0.06 (h^−1^)	3.6 * 20.7	0.73 * 0.93
	6.5	1.80	231	0	0	—	—	—
	11.9	0.40	28	0	0	—	—	—
	11.8	0.40	278	0	0	—	—	—
	11.8	1.80	28	0	0	—	—	—
	11.8	1.80	259	0	0	—	—	—
PAN-S-C	2.0	0.46	24	32.3	16.7	0.314	16.8	0.99
	2.0	0.46	247	47.1	252.7	0.8	250.0	1
	2.0	1.86	24	92.0	11.9	0.923	11.9	0.99
	2.0	1.84	256	80.9	112.7	0.792	112.6	1
	6.0	0.47	25	22.3	11.9	0.472	12.2	0.97
	6.8	0.40	252	42.0	57.4	0.303	57.5	1
	6.1	1.90	25	40.2	5.3	4.163	5.3	1
	6.8	1.84	252	47.2	64.5	0.343	64.5	1
	12.0	0.40	25	0	0	—	—	—
	12.1	0.40	258	0	0	—	—	—
	12.0	1.85	28	25.7	3.9	94.413	3.4	0.99
	12.1	1.80	244	0	0	—	—	—
PAN-C-Act	1.9	0.45	24	100.0	52.8	0.226	52.6	0.99
	1.9	0.47	285	99.9	602.3	0.026	625.0	1
	1.9	1.86	24	100.0	12.9	29.954	12.9	1
	1.9	1.89	285	99.0	149.1	0.748	149.3	1
	6.1	0.46	25	99.6	53.6	0.161	53.2	0.99
	6.7	0.49	246	100.0	505.0	0.800	500.0	1
	6.1	1.86	25	99.8	13.4	6.300	13.3	1
	6.7	1.88	246	100.0	130.7	0.593	129.9	1
	11.9	0.47	25	99.1	52.8	1.191	52.9	1
	12.0	0.48	265	100.0	549.5	0.324	555.6	1
	11.9	1.85	25	99.8	13.5	0.898	13.4	1
	12.0	1.90	265	100.0	139.4	5.184	138.9	1

**Table 3:** Experimental conditions essayed and parameters of the intra-particle diffusion model for red wood dye adsorption with carbons prepared.

Sample	Experimental Conditions			Intra-particle-diffusion Model					
	pH	S/L (g L^−1^)	C_i_ (mg L^−1^)	First Stage			Second Stage		
				K_1d_ (mg g^−1^ h^−0.5^)	I_1_ (mg g^−1^)	R^2^	K_2d_ (mg g^−1^ h^−0.5^)	I_2_ (mg g^−1^)	R^2^
CAC	2.0	0.53	24	61.35	18.24	0.50	0.06	44.78	0.03
	2.0	0.44	250	503.67	190.16	0.55	6.02	516.29	0.79
	2.0	1.76	24	16.53	5.63	0.44	0.11	12.45	0.41
	2.0	1.91	250	176.73	59.34	0.44	0.12	128.92	0.02
	6.1	0.5	25	80.07	20.36	0.58	0.06	54.34	0.14
	6.5	0.46	234	553.11	66.56	0.85	25.43	362.05	0.67
	6.0	1.75	24	19.001	5.47	0.47	0.04	13.23	0.59
	6.5	1.74	236	175.85	59.12	0.44	0.05	130.55	0.01
	12.6	0.43	25	79.60	16.14	0.54	0.67	45.06	0.19
	12.1	0.45	259	317.18	114.40	0.76	24.67	371.74	0.77
	12.6	1.74	26	71.14	16.97	0.61	0.86	44.12	0.29
	12.1	1.78	260	378.18	98.76	0.80	24.70	371.14	0.77
PAN-C	2.0	0.44	24	5.51	1.23	0.57	0.51	2.15	0.93
	2.3	0.45	256	89.38	10.25	0.84	6.49	21.47	0.90
	2.0	1.70	24	47.13	8.62	0.67	3.06	26.87	0.99
	2.0	1.87	256	20.32	2.94	0.95	0.10	20.62	0.03
	6.0	0.44	24	0.28	0.01	0.96	0.02	0.35	0.92
	6.5	0.48	231	—	—	—	—	—	—
	6.1	1.87	24	0.47	0.27	0.85	—	—	—
	6.5	1.8	231	—	—	—	—	—	—
	11.9	0.4	28	—	—	—	—	—	—
	11.8	0.4	278	—	—	—	—	—	—
	11.8	1.8	28	—	—		—	—	—
	11.8	1.8	259	—	—	—	—	—	
PAN-S-C	2.0	0.46	24	11.68	3.77	0.62	1.28	10.35	0.93
	2.0	0.46	247	355.72	93.88	0.52	0.72	249.18	0.99
	2.0	1.86	24	13.22	1.05	0.96	0.47	10.80	0.72
	2.0	1.84	256	258.27	41.39	0.42	1.08	107.45	0.94
	6.0	0.47	25	8.04	1.62	0.91	—	—	—
	6.8	0.4	252	89.38	0.76	0.99	11.15	43.40	0.56
	6.1	1.90	25	9.04	0.31	0.93	0.73	4.25	0.73
	6.8	1.84	252	102.09	4.50	0.88	1.07	58.28	0.63
	12.0	0.4	25	—	—	—	—	—	—
	12.1	0.4	258	—	—	—	—	—	—
	12.0	1.85	28	3.49	1.01	0.67	0.02	3.54	0.04
	12.1	1.8	244	68.53	14.13	0.63	0.03	44.08	0.09
PAN-C-Act	1.9	0.45	24	44.81	23.55	0.38	0.41	49.65	0.89
	1.9	0.47	285	736.27	168.26	0.56	12.85	529.11	0.60
	1.9	1.86	24	11.39	6.01	0.38	0.02	12.83	0.84
	1.9	1.89	285	181.03	60.86	0.37	0.59	145.57	0.65
	6.1	0.46	25	48.72	23.48	0.42	0.15	51.4	0.12
	6.7	0.49	246	351.37	223.0	0.48	1.93	515.53	0.27
	6.1	1.86	25	16.28	5.8	0.34	0.01	13.28	0.04
	6.7	1.88	246	115.07	58.85	0.39	0.20	129.25	0.49
	11.9	0.47	25	65.86	23.11	0.35	0.09	52.48	0.32
	12.0	0.48	265	552.79	274.65	0.25	0.28	560.43	0.30
	11.9	1.85	25	16.12	5.83	0.34	0.09	12.92	0.34
	12.0	1.90	265	140.21	69.01	0.25	0.01	139.09	0.47

**Table 4:** Reported dye adsorption capacities of azo dyes on commercial activated carbons.

Supplier	Dye	*q_max_* (mg g^−1^)	Source
PAN-C	Red GRA 200%	70.5	This study
PAN-S-C		252.7	
PAN-C-Act		602.3	
CAC		556.1	
Chemviron F-400	Remazol Golden Yellow	714	[36]
	Remazol Red	278	
	Remazol Black B	213	
Merck	Reactive Red 120 (RR-120)	267	[36]
	Reactive Violet f	517	[37]
Panreac	Mordant Blue 9	213	[38]
Fagron		221	
Norit Darco	Reactive Black 5	564	[39]
Norit R008		796	
Norit PK		474	
Filtrasorb Corp E-400	Acid Yellow 117	156	[40]
	Reactive Red	112	[41]
Calgon Corp F-400	Direct Brown 1	8	[42]

## Data Availability

The raw data supporting the conclusions of this article will be made available by the authors on request.
